# Integrating Strategies of Herbal Metabolomics, Network Pharmacology, and Experiment Validation to Investigate Frankincense Processing Effects

**DOI:** 10.3389/fphar.2018.01482

**Published:** 2018-12-18

**Authors:** Zhangchi Ning, Chun Wang, Yuanyan Liu, Zhiqian Song, Xinling Ma, Dongrui Liang, Zhenli Liu, Aiping Lu

**Affiliations:** ^1^Institute of Basic Theory for Chinese Medicine, China Academy of Chinese Medical Sciences, Beijing, China; ^2^School of Chinese Materia Medica, Beijing University of Chinese Medicine, Beijing, China; ^3^School of Chinese Medicine, Hong Kong Baptist University, Hong Kong, China

**Keywords:** processing, integrating strategy, herbal metabolomics, intestinal absorption effect, pharmacological effect

## Abstract

In-depth research on processing can promote the globalization of processed herbs. The purpose of this study is to propose an improved strategy for processing effect investigation. Frankincense and processed frankincense were used as research subjects. First, high-speed countercurrent chromatography (HSCCC) and preparation high-performance liquid chromatography (PHPLC) techniques were used for major compounds isolation and minor compounds concentration. Processed frankincense was subjected to two stepwise solvent systems, namely, *n*-hexane:ethanol:water (6:5:1) and *n*-hexane:methyl-acetate:acetonitrile:water (4:4:3:4), to yield 12 fractions, and 18 compounds were further separated. Second, a comprehensive metabolomic analysis conducted by ultrahigh-performance liquid-chromatography/electrospray-ionization mass spectrometry (UHPLC-Qtof-MS) coupled with multivariate statistics was performed to fully characterize the chemical components and discover the potential biomarkers between frankincense and processed frankincense. In total, 81 metabolites, including the 18 separated compounds, were selected as potential biomarkers between frankincense and processed frankincense among 153 detected compounds for their VIP values of greater than one. The tirucallane-type compounds and components with 9,11-dehydro structures clearly occurred at high levels in the processed frankincense, while lupine-type compounds and those with 11-keto structures were significantly higher in frankincense. Then, a network pharmacology model was constructed to decipher the potential mechanisms of processing. Intestinal absorption properties prediction indicated the possibility of processing-related absorption enhancement. A systematic analysis of the constructed networks showed that the C-T network was constructed with 18 potential biomarkers and 69 targets. TNF and IL-1β were among the top-ranked and were linked by 8 and 7 pathways, which were mainly involved in inflammation. The arachidonic acid metabolism pathway exhibited the highest number of target connections. Finally, the prediction was validated experimentally by an intestinal permeability and efficacy assay. The experiments provided convincing evidence that processed frankincense harbored stronger inhibition effects toward TNF-α-, IL-1β- and arachidonic acid-induced platelet aggregation. The processing procedure leads to changes of the chemical metabolites, which triggers the enhancement of absorption and cure efficiency. The global change of the metabolites, absorption and pharmacological effects of processing were depicted in a systematic manner.

## Introduction

Herbal medicine is attracting increasing attention and acceptance in the world due to its special contribution to the treatment of chronic diseases. The processing of herbal medicine, called Pao Zhi, is a unique part of traditional Chinese medicine (TCM) and has been widely used in the preparation of Chinese materia medica for thousands of years. It can alter the properties and functions of remedies, increase medical potencies, and reduce the toxicities and side effects of raw herbs (Zhao et al., 2015). In the Chinese Pharmacopoeia (2015 edition), raw herbs and processed herbs are all listed as specific items. However, in the Japanese (Japanese Pharmacopoeia Commission, 2011), North American (The United States Pharmacopeia Convention, 2013), British (British Pharmacopoeia Commission, 2001), and European pharmacopoeias (European Pharmacopoeia Commission, 2013), only raw herbs are listed. The reason for limiting the globalization of Pao Zhi is the lack of accurate information about bioactive compounds and the absorption and pharmacological mechanisms between raw and processed herbs. Until now, researchers have tried to illustrate the effects of processing from the perspective of chemical research and absorption and pharmacological activity enhancement. However, the systematic relationship between the changes of compounds and the absorption and cure efficiencies were ignored.

Chemical studies of processing provided useful information for understanding chemical changes. However, the identification and evaluation of the changes of compounds during processing are hampered by the content differences among complex components in herbal medicines and the limitations of analytical methods. This generates a critical requirement for appropriate methods to reveal definitive information. Additionally, phytochemical analysis is strongly dependent on efficient separation methodologies that are scalable to the preparative scale due to the need for bioactivity evaluation. Metabolomics is an ‘omics’ approach that aims to comprehensively analyze all metabolites in a biological sample (Zhang et al., 2010). However, the approach failed to provide higher peak capacity from a single dimensional separation. High-speed countercurrent chromatography (HSCCC) is a technique based on the partitioning of compounds between immiscible liquid phases. It seems to be beneficial in yielding a larger preparative recovery due to the minimized chemosorptive effects ascribed to this methodology. A strategy of HSCCC combined with on-line high-performance liquid chromatography-mass spectrometry (HSCCC-HPLC-MS) has been applied for the isolation and characterization of plant components (Grace et al., 2014). It successfully extended the peak capacity. It also resulted in lower detection limits and provided a selective and efficient method for the isolation and characterization of chemical compounds (Grace et al., 2014). The possibility of isomer identification was slim. The nuclear magnetic resonance (NMR) method is immensely significant for structural elucidation, particularly of complex molecules with chiral centers (Biais et al., 2009). In the present study, comprehensive metabolites identification combined with bioactivity evaluation was proposed to provide global insight into the complex processing effects of frankincense. In addition to the chemical investigation, the mechanism of processing needs to be further clarified from a global synergistic perspective. However, traditional experimental methods did not fully reveal the comodule association mechanisms of multiple components and multiple targets. In recent years, network pharmacology has emerged as a holistic and efficient tool to decode the underlying mechanisms of multitarget herbs by analyzing various networks of complex and multilevel interactions (Zhang Y. et al., 2015). Here, we established a novel platform for researching the processing mechanisms of frankincense by a metabolomics-based network pharmacology method integrated with both *in vitro* and *in vivo* bioavailability evaluations of the multitarget treatment strategy.

Frankincense (Ruxiang in Chinese), the gum resin of *Boswellia sacra* Flück. (*B. carteri* Birdw), *B. papyrifera* (Del.) Hochst., or *B. serrata* Roxb, has been used as a traditional medicine in many countries (Zhang C. et al., 2015). Noteworthy scientific discoveries revealed that the triterpenoid metabolites in frankincense were widely used as anti-inflammatory and anti-cancer agents. However, its efficient *in vivo* drug delivery is often hampered by biological barriers (Du et al., 2015). China is the largest market for frankincense in the world, mainly due to the use of this material in TCM. Only processed frankincense (Cu-Ruxiang in Chinese) can be taken orally, and the raw frankincense can be used externally. It is widely believed that the processing procedure enhances the bioavailability and curative efficacy of frankincense. Research showed that the observed improvement of anticoagulation effects from processed frankincense may result from the increased absorption and bioavailability of triterpenoids (Pan et al., 2015). This result can be seen as a promising direction in disease therapy. To gain insight into the effects of the processing procedure and to further elucidate the mechanisms of the pharmacological effects, it is of significance to develop a fast and comprehensive method for the analysis and preparation of triterpenoid metabolites in frankincense. In the case of the triterpenoid metabolites in frankincense, routine analytical methods are not suitable for separation due to the existence of compositions with great disparity of content and containing a large number of isomers.

In this paper, an improved strategy is put forward to investigate processing effects of frankincense. Principal compounds separation and minor concentrated metabolites enrichment from frankincense and processed frankincense were conducted separately by HSCCC and PHPLC techniques for larger lab-scale preparation and subsequent structural identification. A comprehensive analysis including lower-concentration metabolites was conducted by UHPLC-Qtof-MS to entirely characterize the chemical components and to discover the potential biomarkers between frankincense and processed frankincense. Then, a network pharmacology screening was developed to predict the effect of processing. Finally, the prediction was validated experimentally through an intestinal permeability and efficacy assay. The global changes of the metabolites and the intestinal absorption and pharmacological effects of processing were supposed to be depicted in a systematic manner.

## Materials and Methods

### Chemicals and Materials

In total, five batches frankincense were purchased from Beijing Tongrentang Medicine Corporation, Ltd. (Beijing, China), Beijing Ben Cao Fang Yuan Medicine Corporation, Ltd. (Beijing, China) and Beijing Qiancao Traditional Chinese Medicine Corporation, Ltd. (Beijing, China). Their plant species were identified to belong to *Boswellia papyrifera* according to the literature (Zhang C. et al., 2015). Rice vinegar was obtained from Beijing Er Shang Long He Food Co., Ltd. (Beijing, China). HPLC-grade acetonitrile, methanol, formic acid and ammonium formate were obtained from Dikma Technologies Inc. (Beijing, China). Deionized water (18.2 MΩ) was obtained from a Milli-Q water purification system (Millipore, Bedford, MA, United States). *N*-hexane, acetonitrile, methyl acetate, and methanol used for active fraction preparation and HSCCC separation were of analytical grade and purchased from Sinopharm Chemical Reagent Co., Ltd. (Shanghai, China). U937 cells were from the Pasture Institute of Iran. FBS, RPMI-1640 medium, fetal bovine serum and penicillin/streptomycin were obtained from Gibco (MA, United Kingdom). The Cell Counting Kit-8 (CCK-8) was purchased from Dojindo (Kumamoto, Japan). Lipopolysaccharide (LPS) and propylene glycol methyl ether acetate (PMA) were purchased from Sigma-Aldrich (St. Louis, MO, United States). The Assay Design Enzyme-Linked Immunosorbent Assay kit (ELISA) was obtained from Beijing 4A Biotech Co., Ltd. (Beijing, China). 3-Acetyl-11-keto-β-boswellic acid (AKBA) was purchased from the National Institute for Control of Biological and Pharmaceutical Products of China (Beijing, China). 11-Keto-β-boswellic acid (KBA), α-boswellic acid (α-BA), β-boswellic acid (β-BA), 3-acetyl-α-boswellic acid (α-ABA), and 3-acetyl-β-boswellic acid (β-ABA) were purchase from ChromaDex Int. (Irvine, CA, United States). Tsugaric acid A and β-elemolic acid were from Extrasynthese (Lyon, France). β-Elemonic acid was obtained from Chengdu DeSiTe Biological Technology Co., Ltd. (Chengdu, China).

### Frankincense Processing

The Al-F_2_11AE induction cooker (ASD Corporation, Zhejiang, China) equipped with temperature control and time programming functions was used for processing. The processed frankincense was prepared by stir-frying with rice vinegar according to the procedure recorded in the Chinese Pharmacopoeia (2015 edition). Briefly, 200 g frankincense was put into a heated pot at the temperature 240°C, stir-fried quickly for 9 min and sprayed with 20 mL rice vinegar [this component has been investigated (Ning et al., 2017a)] during the next 2 min until the surface became glossy (Ning et al., 2017b). All the raw and processed samples were vacuum-dried and pulverized into a fine powder using an 80-mesh sieve.

### Preparation of Samples

The processed sample powder (1 kg) was extracted with 30.0 L methanol under sonication for 30 min. After filtration and evaporation, the methanol extraction (540.05 g) was obtained with a yield of 54.05%.

### HSCCC Separation

#### Preparation of the Three-Phase Solvent System and Sample Solutions

A three-phase solvent system for HSCCC separation composed of *n*-heptane:methyl acetate:acetonitrile:water (4:4:3:4, v/v/v/v) was thoroughly mixed in a separatory funnel and allowed to stand until three clear layers were formed at 25°C. Then, the upper phase, middle phase and lower phase were separated. The sample solution was prepared by dissolving 1.0 g of the methanol extraction in 5 mL of the middle phase.

#### Measurement of Partition Coefficients (*K*)

The upper phase (2 mL) and the middle phase (2 mL) were delivered in glass test tubes in which 10 mg of methanol extraction was added, mixed thoroughly, and allowed to stand until two clear layers were formed. Each phase solution (5 μL) was determined by an Agilent 1200 HPLC apparatus (Agilent Technologies Inc., Palo Alto, CA, United States). HPLC separation was performed on an Agilent XDB-C_18_ column (4.6 mm × 50 mm, 1.8 μm) using a mobile phase of methanol and water (containing 0.1% formic acid) at a flow rate of 1.0 mL/min. The gradient program [MeCN:H_2_O (containing 0.1% phosphoric acid), v/v] was 60:40 (*t* = 0 min), 60:40 (*t* = 30 min), 80:20 (*t* = 35 min), 80:20 (*t* = 45 min), and 85:15 (*t* = 60 min). The peak areas of the upper phase and the middle phase were recorded as A_upper_ and A_lower_, respectively. The partition coefficients (*K*) of KBA, AKBA, α-BA, β-BA, α-ABA, and β-ABA were obtained by the following equation: *K_D_* = A_upper_/A_lower_.

#### HSCCC Separation of Samples

The head-to-tail mode was applied for HSCCC separation. The upper phase was filled into the multilayer coiled columns of the TBE-300 HSCCC apparatus (Tauto Biotechnique Company, Shanghai, China as the stationary phase.) by an ÄKTA Purifier^TM^ P-900 (General Electric Company, Fairfield, CT, United States) as the stationary phase. The apparatus was then rotated at 800 rpm while the lower phase was pumped into the coil column at a flow rate of 3.0 mL/min. After hydrodynamic equilibrium was reached, the retention rate was determined, and the sample solution was injected into the column. During the separation process, the column temperature was controlled at 25°C. The effluent from the coil-column was monitored by an ÄKTA Purifier^TM^ UV-900 (General Electric Company, Fairfield, CT, United States), and fractions were collected by an ÄKTA Purifier^TM^ Frac-920 (General Electric Company, Fairfield, CT, United States).

### Separation, Purification, and Structural Identification of Compounds

The fractions were further purified by a preparative HPLC apparatus (PHPLC, Agilent 1200s, Agilent Technologies Inc., Palo Alto, CA, United States) equipped with a G13611A prep pump, G2260A prep automatic sampler, G1315D diode array detector, G1364B prep fraction collector and Agilent Zorbax SB-C_18_ column (21.2 mm × 250 mm, 7 μm). The elution was carried out in isocratic mode with different ratios of methanol and water containing 0.1% formic acid at 5 mL/min. The detection wavelengths were 210, 250, and 280 nm. The PHPLC fractions were collected and evaporated to dryness.

The target compounds obtained by HSCCC and PHPLC separation were identified by their UV, MS,^1^ H-NMR and ^13^C-NMR spectra. The NMR spectra were recorded on Bruker AM-400 spectrometers (Bruker, Karlsruhe, Germany) using trimethylsilyl (TMS) as the internal reference.

### Metabolite Profiling of Frankincense and Processed Frankincense

#### UHPLC-Qtof-MS Analysis of the Extractions of Frankincense, Processed Frankincense and HSCCC Fractions

High-resolution mass spectra were measured on an Agilent 6520 Accurate-Mass TOF LC/MS (Agilent Technologies, Santa Clara, CA, United States). UHPLC separation was performed on an Agilent XDB-C_18_ column (4.6 mm × 50 mm, 1.8 μm) using a mobile phase of methanol and water (containing 0.1% formic acid) in the positive mode and of methanol and water (containing 0.2% formic acid and 5 mM ammonium formate) in the negative mode, both at a flow rate of 0.5 mL/min. The gradient programs of the positive and negative modes (MeOH:H_2_O, v/v) were both 70:30 (*t* = 0 min), 85:15 (*t* = 10 min), 90:10 (*t* = 15 min), 92:8 (*t* = 22 min), 95:5 (*t* = 25 min), and 98:2 (*t* = 40 min). The injection volume of each sample was 2 μL. Both positive and negative modes were performed. The ESI conditions were as follows: gas temperature, 300°C; drying gas, 8 L⋅min^-1^ and nebulizer, 20 psi. The TOF conditions were as follows: fragmentor, 200 V; skimmer, 60 V and OCTRFV, 200 V. Accurately weighed powder samples (50 mg) of frankincense or processed frankincense were placed in conical flasks separately and sonicated with 10 mL methanol for 30 min. The supernatant solution was filtered through a 0.22-μm filter membrane. The HSCCC fractions were evaporated to dryness and redissolved by methanol and then filtered through a 0.22-μm filter membrane. Each sample was prepared in triplicate and detected twice using *UHPLC-Qtof-MS*.

#### Data Processing and Multivariate Analysis

The mass data acquired were imported to MassHunter Profinder (version B.06.00) and Mass Profiler Professional (version B.02.00) for peak detection and alignment. The retention time and m/z data for each peak were determined by the software. The parameters were set as follows: the full scan mode was employed in the mass range of 100–600 amu; mass tolerance, 0.1 Da and noise elimination level, 5.

Multivariate statistical analyses, including unsupervised principal component analysis (PCA) and orthogonal partial least-squares-discriminant analysis (OPLS-DA), were performed using the Simca-P (version 13.0) (Sartorius Stedim Biotech, Malmö, Sweden) statistical package to find the potential biomarkers. The significant *p-*value for all analyses in this study was set to 0.05.

The structural identification of potential biomarkers between frankincense and processed frankincense was performed according to the retention time, UV-visible spectra and mass characteristics of the reference substances and separated compounds. Meanwhile, the relationships between the separated compounds and potential biomarkers were considered. The possessed discriminatory power of potential biomarkers was investigated by network pharmacology.

### Prediction of Absorption, Target, and Pathway

Crucial absorption parameters, including integrated oral bioavailability (OB) value and Caco-2 permeability values, were used to predict the absorption properties of the potential biomarkers the possessed discriminatory power. Their absorption parameters were obtained in TCMSP^1^. With respect to absorption predictions of frankincense and processed frankincense having problems with multiple compound attributes, a decision analysis method was proposed. A probability value (*Pij*) and weight index (WI) (Zhou, 2009) of components in the evaluation of absorption was calculated by Eqs. (1) and (2). The values of the OB and Caco-2 permeability of frankincense and processed frankincense were calculated by Eqs. (3) and (4).

(1) $$Pij=\underset{1\leq i\leq m}{\min}Cij/Cij\cdot \sum_{i=1}^{m}\frac{\min_{1\leq i\leq m}Cij}{Cij}$$

(2) $$WI=[1+{\left(\ln m\right)}^{-1}\sum_{i=1}^{m}Pij]/\sum_{i=1}^{m}\left[1+{\left(\ln m\right)}^{-1}\sum_{i=1}^{m}Pij\right]$$

(3) OBJ=WI·OBi·cij

(4) (Caco−2)j=WI·cij(Caco−2)i

Here, *n* is the number of potential biomarkers possessing discriminatory power in *m* number of different frankincense samples. *c_ij_* represents the concentration of compound *j* in *i* kinds of frankincense samples.

To obtain the target of the biomarkers of frankincense and processed frankincense, a similarity ensemble approach (SEA) (Keiser et al., 2007) and PharmMapper server (Gfeller et al., 2014) were employed to identify the targets. All chemical structures were prepared and converted into canonical SMILES using the Open Babel Toolkit (version 2.4.1) (O’Boyle et al., 2011). In addition, the target results were confirmed by literature reviews.

The latest pathway data were extracted from the Kyoto Encyclopedia of Genes and Genomes (KEGG) database (Draghici et al., 2007) for KEGG pathway enrichment analyses. *P*-values were set at 0.05 as the cut-off criterion. These analytical results were annotated by Pathview (Luo and Brouwer, 2013) in the R Bioconductor package^2^.

The component-target (C-T) network was constructed to find the key targets. Then, the target-pathway (T-P) network was established to determine the relationships between the targets and pathways. Cytoscape 3.5.1 (Shannon et al., 2003), an open-source software platform for visualizing complex networks, was used to visualize the networks.

### Validation of Absorption Prediction

#### Rat Everted Gut-Sac Assay

The powders of frankincense and processed frankincense (3 g) obtained using an 80-mesh sieve were individually mixed in 500 mL 1% CMC-Na and ground for 10 min to obtain suspensions. The rat everted gut-sac model was chosen (He et al., 2017). It was prepared from a Sprague Dawley rat intestine. The first 10 cm of the intestine from the stomach was dissected and immediately rinsed with Ringer’s buffer. The intestine was everted in a manner such that the distal end of the segment remained tied to the everting rod. It was then blotted and trimmed to a length of 5.5 cm. The everted gut-sac was filled with 0.5 mL of Ringer’s buffer to maintain the same osmotic pressure against the outside solution using 1 mL of a syringe fitted with a blunt needle. The everted gut-sac was tied shut on both ends of the intestine. It was then placed and incubated in 30 mL Erlenmeyer flasks containing 25 mL of Ringer’s buffer, frankincense or processed frankincense suspensions. Throughout the experiment, 95% oxygen and 5% CO_2_ were continuously bubbled through the incubation buffer. The inner solution of the everted gut-sac was collected using a syringe fitted with a needle. An accurately measured 3 mL aliquot of the inner solution of the everted gut-sac and 10 mL ethyl acetate was added. The mixture was extracted three times. The organic layer was collected, dried by nitrogen, redissolved in 100 μL methanol and tested by ultrahigh-performance liquid chromatography coupled with triple-quadrupole tandem mass spectrometry (UHPLC-QqQ-MS) for compound bioavailability investigation. This study was approved by the Research Ethics Committee of Institute of Basic Theory of Chinese Medicine, China Academy of Chinese Medical Sciences.

#### UHPLC-QqQ-MS/MS Conditions

An Agilent 1260 UHPLC/6410 QqQ system (Santa Clara, CA, United States) equipped with an electrospray ionization (ESI) source (data analysis software Masshunter version B.01.04) was utilized for determination of the inner solution of the everted gut-sac. The negative ion mode was chosen. The nitrogen temperature was kept at 300°C, the flow rate at 11 L/min, nebulizer at 15 psi, and the capillary voltage at 4,000 V.

UHPLC condition was carried out on an Agilent XDB-C_18_ column (4.6 × 100 mm, 1.8 μm) (0.5 mL/min) using a mobile phase of water (containing 0.1% formic acid and 10 mM ammonium formate) and acetonitrile with gradient elution. The gradient program (MeCN/H_2_O, v/v) was 85:15 (*t* = 0 min), 85:15 (*t* = 20 min), 98:2 (*t* = 35 min), and 98:2 (*t* = 40 min). Each sample was prepared in triplicate. The injection volume of each sample was 2 μL. Bioavailability was evaluated by the uptake rate of the compounds. Rate of uptake = *C*_innersolutionofevertedgut-sac_/*C*_frankincenseorprocessedfrankincensesuspensions_ × 100%.

### Validation of Pharmacological Prediction

#### Anti-inflammation Assay

Human leukemic U937 cells were cultured in RPMI-1640 medium with 10% fetal bovine serum and 1% penicillin/streptomycin. They were placed into a 96-well cell culture plate for cell viability assay using CCK-8. For this, 100 ng/mL PMA was added. Then, the cells were washed with PBS, followed by incubation with methanol extract of frankincense or processed frankincense (2–100 μg/mL) for up to 48 h at 37°C in 5% CO_2_. Next, 10 μL CCK-8 was added to incubate for another 3 h. For the validation assay, cells were seeded into a 12-well cell culture plate at a density of 1 × 10^6^ cells/well and incubated with 100 ng/mL PMA for 48 h in a 5% CO_2_ incubator. Then, they were washed twice, stimulated by LPS and treated with or without frankincense and processed frankincense extracts at 8 μg/mL and 16 μg/mL and validated by CCK-8 assay for 48 h. Cell supernatants were harvested. TNF-α and IL-1β production were detected using an ELISA kit. All data were expressed as the mean ± standard deviation (SD). The statistical significance was evaluated using SPSS version 22.0 (SPSS, Cary, NC, United States).

#### Platelet Aggregates Assay

For the arachidonic acid (AA)-induced platelet aggregation assay, male rats were anesthetized with chloral hydrate (300 mg/kg). The blood drawn from the abdominal aortas was anticoagulated with frankincense and processed frankincense extracts (16 μg/mL) and heparin (20 U/mL). All platelet aggregation studies were performed using a Chrono-log platelet aggregometer (Chrono-log Co., United States). This system measures the increase in impedance caused by platelet aggregates between two pairs of electrodes, enabling two simultaneous measurements for duplicate analysis. Single-use cuvettes containing a Teflon-coated stirrer (800 rpm) were filled with prewarmed 500 μL physiologic saline and 500 μL whole blood. After 10 min of incubation, tests were initiated by adding a stimulating agonist to the test cell. In our study, we used AA (0.5 mM) as the agonist. Aggregation was recorded for 6 min, and the results were given in the maximum aggregation and area under the curve (AUC). AUC (aggregation × 6 min) in units and were used for further analysis.

## Results and Discussion

### Principal Compounds Separation and Minor Compounds Concentration

#### Optimization of the Solvent System and Conditions for HSCCC

A suitable solvent system is essential for the satisfactory separation of HSCCC (Costa et al., 2015). Ito (Lee et al., 1990) proposed a two-phase solvent system composed of *n*-hexane, ethanol, and water with a volume ratio of 6:5:1 for the separation of triterpenoid acids from frankincense. Considering the high separation capacity of the three-phase solvent system for various compounds (Yanagida et al., 2007), a series of three-phase solvent systems was formed at various volume ratios. As shown in Supplementary Table S1, several three-phase solvent systems and two-phase solvent systems were optimized.

The selection of an appropriate three-phase solvent system was performed by calculating the *K_D_* values of the compounds. According to the dissolution status of the methanol extract in the biphasic layer, the *K_D_* values were calculated between two immiscible layers of six main peaks, including KBA, AKBA, α-BA, β-BA, α-ABA, and β-ABA, in the HPLC chromatogram (detailed data can be found in the Supplementary Material). The range of 0.5 ≤*K*_D_ ≤ 2 and the ratio of *K*_Dx_/*K*_Dy_ ≥ 1.5 (*K*_Dx_ and *K*_Dy_ are the partition coefficient values of adjacent peaks) are required for effective HSCCC separation (Oka et al., 1991). After comparing the *K*_D_ values, a three-phase solvent system composed *n*-heptane:methyl acetate:acetonitrile:water at a ratio of 4:4:3:4 was chosen.

The rotating speed (600 rpm, 800 rpm, and 1,000 rpm) and flow rate (2 mL/min, 3 mL/min, and 5 mL/min) of the mobile phase, along with the separation temperature (20°C, 25°C, and 30°C), were the main factors influencing the separation. Under the conditions of the same flow rate (3 mL/min) and the same separation temperature (25°C), the rotating speed of 800 rpm was proven to realize the best separation. The flow rate of 3 mL/min exhibited the best retention rates of the stationary phase, and the separation temperature was settled at room temperature (25°C). All optimized conditions were applied for HSCCC separation of the methanol extracts.

#### HSCCC Separation of Processed Frankincense

**FIGURE 1:**
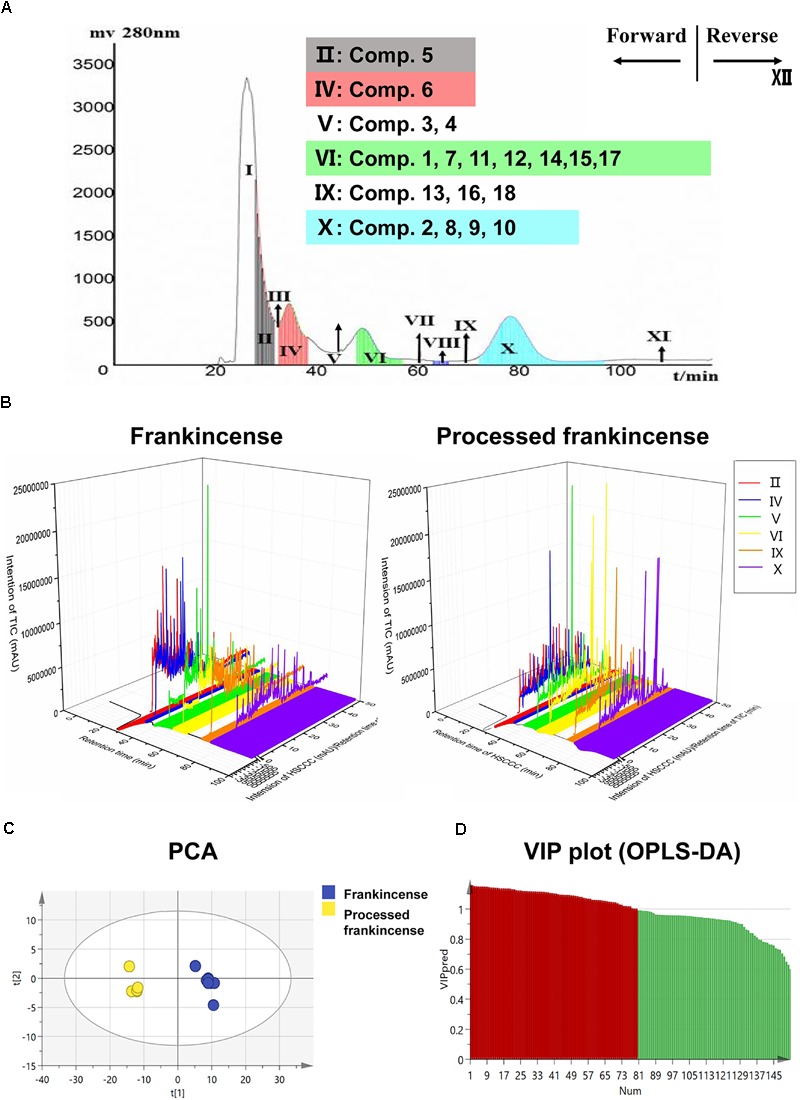
Metabolomics research of frankincense and processed frankincense. **(A)** The application of HSCCC for partial analysis for a larger lab-scale preparation. **(B)** Information extension of the HSCCC and UHPLC-Qtof-MS combination. **(C)** PCA score plots of frankincense and processed frankincense. **(D)** Variable importance plot (VIP) of the OPLS-DA model between frankincense and processed frankincense.

The HSCCC separation chromatogram of processed frankincense is shown in Figure 1A. The retention rate of the stationary phase was 64.7%. Twelve HSCCC fractions were obtained and analyzed by HPLC. The results showed that HSCCC fraction II contained compound 5; fraction IV contained compound 6; fraction V contained compound 3 and compound 4; fraction VI contained compounds 1, 7, 11, 12, 14, 15, and 17; fraction IX contained compounds 13, 16, and 18; and fraction X contained compounds 2, 8, 9, and 10.

#### Structural Identification of the Separated Compounds

Eighteen compounds were isolated from the methanol extract of processed frankincense.

Compounds 3 and 9 were isolated from HSCCC fractions V and X, respectively. Their UV absorption was 280 nm. ESI-TOF-MS indicated that their molecular ions were located at m/z 453 [M-H]^-^, corresponding to a molecular formula of C_30_H_46_O_3_. Both showed the same signal at *δ* 5.0–6.0 (Schweizer et al., 2000). The compound 3 ^1^H-NMR (CDCl_3_, 400 MHz) results were as follows: *δ* 5.68 (1H, d, *J* = 5.6 Hz), 5.48 (1H, d, *J* = 5.6 Hz), 5.33 (1H, br s), 1.38 (3H, s), 1.19 (3H, s), 1.13 (3H, s), 0.93 (3H, s), 0.86 (3H, s), and 0.8 (3H, d, *J* = 6.3 Hz). The^13^C-NMR (CDCl_3_, 400 MHz) results were: *δ* 35.7 (C-1), 27.2 (C-2), 76.2 (C-3), 56.3 (C-4), 45.7 (C-5), 16.1 (C-6), 32.5 (C-7), 41.9 (C-8), 154.9 (C-9), 38.6 (C-10), 116.1 (C-11), 121.2 (C-12), 145.3 (C-13), 42.7 (C-14), 25.8 (C-15), 27.0 (C-16), 32.8 (C-17), 45.6 (C-18), 46.8 (C-19), 31.2 (C-20), 34.1 (C-21), 36.0 (C-22), 12.5 (C-23), 181.0 (C-24), 20.0 (C-25), 21.2 (C-26), 24.5 (C-27), 26.7 (C-28), 26.9 (C-29), and 27.2 (C-30). The compound 9 ^1^H-NMR (CDCl_3_, 400 MHz) results were as follows: *δ* 5.68 (1H, d, *J* = 5.6 Hz), 5.48 (1H, d, *J* = 5.6 Hz), 5.33 (1H, br s), 1.38 (3H, s), 1.16 (3H, s), 1.11 (3H, s), 1.03 (3H, s), 0.93 (3H, s), 0.86 (3H, s), and 0.8 (3H, s). The ^13^C-NMR (CDCl_3_, 400 MHz) results were: *δ* 35.3 (C-1), 26.4 (C-2), 75.3 (C-3), 54.3 (C-4), 43.8 (C-5), 16.3 (C-6), 34.7 (C-7), 41.2 (C-8), 156.3 (C-9), 36.3 (C-10), 119.2 (C-11), 123.3 (C-12), 143.2 (C-13), 41.9 (C-14), 27.3 (C-15), 28.3 (C-16), 34.2 (C-17), 58.1 (C-18), 38.2 (C-19), 38.9 (C-20), 28.6 (C-21), 42.7 (C-22), 13.2 (C-23), 179.3 (C-24), 18.2 (C-25), 22.1 (C-26), 24.8 (C-27), 26.9 (C-28), 15.4 (C-29), and 23.8 (C-30). Compound 9 gave a longer retention time in HPLC analysis than did compound 3. This result was agreed with the conclusion that β-configurations always give longer retention times than do α-configurations. Therefore, compound 3 was identified as 9,11-dehydro-α-bowellic acid, and compound 9 was identified as 9,11-dehydro-β-bowellic acid (as shown in Figure 1A). The chemical structures were identified on the basis of a reference (Schweizer et al., 2000).

Compound 4 was isolated from HSCCC fraction V, and compound 10 was isolated from fraction X. Their UV absorption was 280 nm. ESI-TOF-MS indicated that their molecular ions were located at m/z 495 [M-H]^-^, corresponding to a molecular formula of C_32_H_48_O_4_. The compound 4 ^1^H-NMR (CDCl_3_, 400 MHz) results were as follows: *δ* 5.67 (1H, d, *J* = 5.6 Hz, H-11), *δ* 5.49 (1H, d, *J* = 5.6 Hz, H-12), *δ* 5.33 (1H, br s, H-3), *δ* 2.08 (3H, s OCOCH_3_), 1.32 (3H, s), 1.18 (3H, s), 0.93 (3H, s), 0.89 (3H, s), and 0.81 (3H, s). The ^13^C-NMR (CDCl_3_, 400 MHz) results were: *δ* 35.9 (C-1), 25.9 (C-2), 76.5 (C-3), 53.0 (C-4), 45.9 (C-5), 15.8 (C-6), 32.5 (C-7), 41.9 (C-8), 154.9 (C-9), 38.4 (C-10), 116.1 (C-11), 121.2 (C-12), 145.3 (C-13), 42.7 (C-14), 25.8 (C-15), 27.0 (C-16), 32.8 (C-17), 45.6 (C-18), 46.8 (C-19), 31.2 (C-20), 34.1 (C-21), 36.0 (C-22), 12.7 (C-23), 181.0 (C-24), 20.0 (C-25), 21.2 (C-26), 24.5 (C-27), 26.7 (C-28), 26.9 (C-29), 25.3 (C-30), 170.2 (C-31), and 21.9 (C-32). The compound 10 ^1^H-NMR (CDCl_3_, 400 MHz) results were as follows: *δ* 5.67 (1H, d, *J* = 5.6 Hz, H-11), *δ* 5.49 (1H, d, *J* = 5.6 Hz, H-12), *δ* 5.33 (1H, br s, H-3), *δ* 2.08 (3H, s OCOCH_3_), 1.30 (3H, s), 1.25 (3H, s), 1.18 (3H, s), 0.98 (3H, s), 0.93 (3H, s), 0.89 (3H, s), and 0.81 (3H, s). The ^13^C-NMR (CDCl_3_, 400 MHz) results were: *δ* 33.69 (C-1), 24.33 (C-2), 76.67 (C-3), 57.37 (C-4), 46.9 (C-5), 17.4 (C-6), 33.19 (C-7), 41.34 (C-8), 152.5 (C-9), 39.03 (C-10), 116.56 (C-11), 123.05 (C-12), 141.63 (C-13), 43.39 (C-14), 26.22 (C-15), 28.27 (C-16), 31.86 (C-17), 47.44 (C-18), 39.42 (C-19), 39.06 (C-20), 28.71 (C-21), 40.66 (C-22), 23.25 (C-23), 181.45 (C-24), 19.53 (C-25), 21.19 (C-26), 23.67 (C-27), 31.20 (C-28), 21.77 (C-29), 21.50 (C-30), 170.20 (C-31), and 21.50 (C-32), The α and β isomers were differentiated according to the number of angular methyl signals. The compound 4 was identified as 3-acetyl-9,11-dehydro-α-bowellic acid and compound 10 was identified as 3-acetyl-9,11-dehydro-β-bowellic acid (as shown in Figure 1A). The chemical structures were identified on the basis of a reference (Schweizer et al., 2000).

Compound 13 was isolated from HSCCC fraction IX. ESI-TOF-MS indicated a molecular ion at m/z 497 [M-H]^-^, corresponding to a molecular formula of C_32_H_50_O_4_. The UV absorption was 210 nm. The ^1^H-NMR (CDCl3, 400 MHz) results were as follows: δ 5.23 (1H, s, H-24), δ 4.41 (1H, s, H-3), 2.04 (3H, s, OCOCH_3_), 1.75 (3H, s), 1.64 (3H, s), 1.01 (3H, s), 0.93(3H, s), 0.91 (3H, s), and 0.82 (3H, s). The ^13^C-NMR (CDCl_3_, 400 MHz) results were: *δ* 35.2 (C-1), 22.2 (C-2), 82.1 (C-3), 38.4 (C-4), 51.4 (C-5), 17.8 (C-6), 27.5 (C-7), 135.1 (C-8), 134.8 (C-9), 37.5 (C-10), 20.8 (C-11), 28.4 (C-12), 44.8 (C-13), 50.2 (C-14), 30.2 (C-15), 27.9 (C-16), 48.2 (C-17), 16.4 (C-18), 17.5 (C-19), 47.6 (C-20), 84.8 (C-21), 33.5 (C-22), 26.3 (C-23), 132.4 (C-24), 133.1 (C-25), 20.8 (C-26), 18.6 (C-27), 22.4 (C-28), 20.4 (C-29), 25.1 (C-30), 171.2 (C-31), and 19.1 (C-32). The compound was identified as 3β-acetoxy-5α-lanosta-8,24-dien-21-acid (as shown in Figure 1A) according to the references (Lin et al., 1997; Ko et al., 2008).

Compound 15 was isolated from HSCCC fraction VI. Its ESI-TOF-MS indicated a molecular ion at m/z 455 [M-H]^-^, corresponding to a molecular formula of C_30_H_48_O_3_. The UV absorption was 210 nm. The ^1^H-NMR (CDCl_3_, 400 MHz) results were as follows: *δ* 5.25 (1H, s, H-7), 5.10 (1H, s, H-24), 3.50 (1H, s, H-3), 1.75 (3H, s), 1.70 (3H, s), 1.53 (3H, s), 0.97 (3H, s), 0.93 (3H, s), 0.90 (3H, s), and 0.75 (3H, s). The ^13^C-NMR (CDCl_3_, 400 MHz) results were: *δ* 31.2 (C-1), 25.3 (C-2), 76.5 (C-3), 41.2 (C-4), 44.5 (C-5), 23.9 (C-6), 118.2 (C-7), 145.7 (C-8), 48.3 (C-9), 38.1 (C-10), 17.5 (C-11), 33.4 (C-12), 43.3 (C-13), 51.1 (C-14), 33.9 (C-15), 30.9 (C-16), 47.1 (C-17), 18.8 (C-18), 21.8 (C-19), 49.7 (C-20), 181.4 (C-21), 32.4 (C22), 27.1 (C-23), 123.6 (C-24), 132.2 (C-25), 17.7 (C-26), 25.7 (C-27), 27.8 (C-28), 21.8 (C-29), and 27.3 (C-30). The compound was identified as 3α-hydroxy-tirucall-7,24-dien-21-oic acid (as shown in Figure 1A). The chemical structure was identified on the basis of a reference (HidayahAl-Zikri et al., 2014).

Compound 16 was isolated from HSCCC fraction IX. The UV absorption was 210 nm. Its ESI-TOF-MS indicated a molecular ion at m/z 497 [M-H]^-^, corresponding to a molecular formula of C_32_H_50_O_4_. The ^1^H-NMR (CDCl_3_, 400 MHz) results were as follows: *δ* 5.25 (1H, s, H-7), *δ* 5.10 (1H, s, H-24), *δ* 4.70 (1H, s, H-3), 2.06 (3H, s, OCOCH_3_), 1.71 (3H, s), 1.58 (3H, s), 0.97 (3H, s), 0.93 (3H, s), 0.91 (3H, s), 0.90 (3H, s), and 0.75 (3H, s). The ^13^C-NMR (CDCl_3_, 400 MHz) results were: *δ* 37.4 (C-1), 26.4 (C-2), 78.9 (C-3), 44.5 (C-4), 52.4 (C-5), 22.4 (C-6), 116.5 (C-7), 146.3 (C-8), 48.1 (C-9), 35.1 (C-10), 22.1 (C-11), 30.2 (C-12), 46.2 (C-13), 51.2 (C-14), 34.9 (C-15), 28.9 (C-16), 49.7 (C-17), 17.5 (C-18), 14.8 (C-19), 49.2 (C-20), 182 (C-21), 33.2 (C-22), 28.4 (C-23), 124.8 (C-24), 132.8 (C-25), 24.9 (C-27), 23.7 (C-28), 24.2 (C-29), 26.2 (C-30), 171.2 (C-31), and 14.2 (C-32). The compound was identified as 3α-acetyloxy-tirucall-7,24-dien-21-oic acid (as shown in Figure 1A). The chemical structure was identified on the basis of a reference (Jolad et al., 1981).

Compound 17 was isolated from HSCCC fraction VI. The UV absorption was 210 nm. Its ESI-TOF-MS indicated a molecular ion at m/z 455 [M-H]^-^, corresponding to a molecular formula of C_30_H_48_O_3_. The ^1^H-NMR (CDCl_3_, 400 MHz) results were as follows: *δ* 4.68 (1H, d, *J* = 2.0 Hz, H-29), *δ* 4.56 (1H, d, *J* = 2.0 Hz, H-29), and *δ* 4.05 (H, s, H-3). The ^13^C-NMR (CDCl_3_, 400 MHz) results were; *δ* 38.1 (C-1), 27.1 (C-2), 75.90 (C-3), 53.0 (C-4), 41.7 (C-5), 20.1 (C-6), 34.9 (C-7), 40.9 (C-8), 50.0 (C-9), 31.7 (C-10), 21.7 (C-11), 25.4 (C-12), 37.9 (C-13), 42.9 (C-14), 26.4 (C-15), 35.6 (C-16), 43.0 (C-17), 48.4 (C-18), 48.0 (C-19), 150.3 (C-20), 29.8 (C-21), 40.0 (C-22), 9.8 (C-23), 181.0 (C-24), 15.8 (C-25), 18.5 (C-26), 15.0 (C-27), 18.5 (C-28), 110.6 (C-29), and 21.7 (C-30). The compound was identified as lupeolic acid (as shown in Figure 1A). The chemical structure was identified on the basis of a reference (Verhoff et al., 2014).

Compound 18 was isolated from HSCCC fraction IX. The UV absorption was 210 nm. Its ESI-TOF-MS indicated a molecular ion at m/z 467 [M-H]^-^, corresponding to a molecular formula of C_32_H_52_O_2_. The ^1^H-NMR (CDCl_3_, 400 MHz) results were as follows: *δ* 4.69 (2H, d, *J* = 2.0 Hz, H-29) *δ* 4.57 (1H, d, *J* = 2.0 Hz, H-29), *δ* 1.68 (3H, s, H-30), and *δ* 2.08 (3H, s, OCOCH_3_). The ^13^C-NMR (CDCl_3_, 400 MHz) results were: *δ* 38.8 (C-1), 26.8 (C-2), 76.84 (C-3). 37.0 (C-4), 55.1 (C-5), 15.9 (C-6), 34.3 (C-7), 40.9 (C-8), 49.9 (C-9), 37.2 (C-10), 19.9 (C-11), 27.6 (C-12), 38.3 (C-13), 43.0 (C-14), 30.0 (C-15), 35.6 (C-16), 43.1 (C-17), 48.4 (C-18), 48.1 (C-19), 152.2 (C-20), 33.7 (C-21), 40.1 (C-22), 21.6 (C-23), 21.2 (C-24), 15.93(C-25), 16.1 (C-26), 14.5 (C-27), 18.2 (C-28), 109.5 (C-29), 21.7 (C-30), 182.1 (C-31), and 19.5 (C-32). The fragmentation rules and NMR spectral characteristics of frankincense metabolites may aid in discovery and identification. This compound was identified as lupeol acetate (as shown in Figure 1A) according to a reference (Verhoff et al., 2014).

Other compounds including 1, 2, 5–8, 11, 12 and 14 were verified using reference substances.

### Metabolomics Study of Frankincense and Processed Frankincense

#### Optimization of Chromatographic Conditions

Chromatographic parameters such as the column type (Agilent SB-C_18_, Agilent XDB-C_18_ and Thermo Accucore RP-MS column), mobile phase composition [MeOH-H_2_O, MeCN-H_2_O, MeOH-H2O (containing 0.2% formic acid and 5 mM ammonium formate)], gradient elution procedure, flow rate of the mobile phase (0.2 mL/min, 0.5 mL/min and 0.8 mL/min) and column temperature (20°C, 30°C, and 40°C) were optimized to obtain the proper separation conditions. This demonstrated that the Agilent XDB-C_18_ (4.6 mm × 50 mm, 1.8 μm) column achieved the best separation, which was proven suitable for the analyses of α-amyrin and β-amyrin.

#### Method Validation

A quality control (QC) sample was produced by mixing equal aliquots of each frankincense sample. The precision was evaluated by injecting the QC sample six times. The reproducibility was determined by analyzing the six replicates of the QC sample within 1 day. A system stability test was carried out by injecting a QC sample every five samples during the whole sample analysis procedure.

The extracted ion chromatographic peaks from five ions including the retention time, signal intensity and mass accuracy were selected for method validation in negative ion mode. The relative standard deviations (RSDs) of the retention time for the precision, reproducibility and system stability were 0.14%∼0.32%, 0.16%∼0.87% and 0.14%∼0.43%. The RSDs of the signal intensity were 2.83%∼3.55%, 3.49%∼4.31% and 3.86%∼4.79%. The RSDs of the mass accuracy were 0.14%∼0.26%, 0.22%∼0.29% and 0.23%∼0.31%.

#### Metabolites Analysis of Frankincense and Processed Frankincense

To investigate the chemical changes that occurred after processing, five batches of frankincense and processed frankincense were analyzed. Holistic chemical profiling and discrimination methods have been developed using UHPLC-Qtof-MS together with chemometric methods such as PCA and OPLS-DA.

The total methanol extracts of frankincense and processed frankincense were analyzed. Meanwhile, the frankincense and processed frankincense HSCCC fractions yields in the Section “HSCCC Separation of Processed Frankincense” were also analyzed. Typical UHPLC chromatograms of frankincense and processed frankincense HSCCC fractions are shown in three-dimensional chromatograms (Figure 1B). The X–Y plane presents the HSCCC chromatograms, and the different colors represent the 12 fractions. The X–Z plane presents the total ion chromatograms of the different fractions. Compared with the 54 detected compounds of the methanol extraction, the HSCCC separation realized detection of 153 compounds. The results showed that compared to the methanol extract, the HSCCC fractions could result in detection of more compounds. The combination of HSCCC and UHPLC-Qtof-MS provides a powerful technique for global chemical profiling of the complex matrix.

**FIGURE 2:**
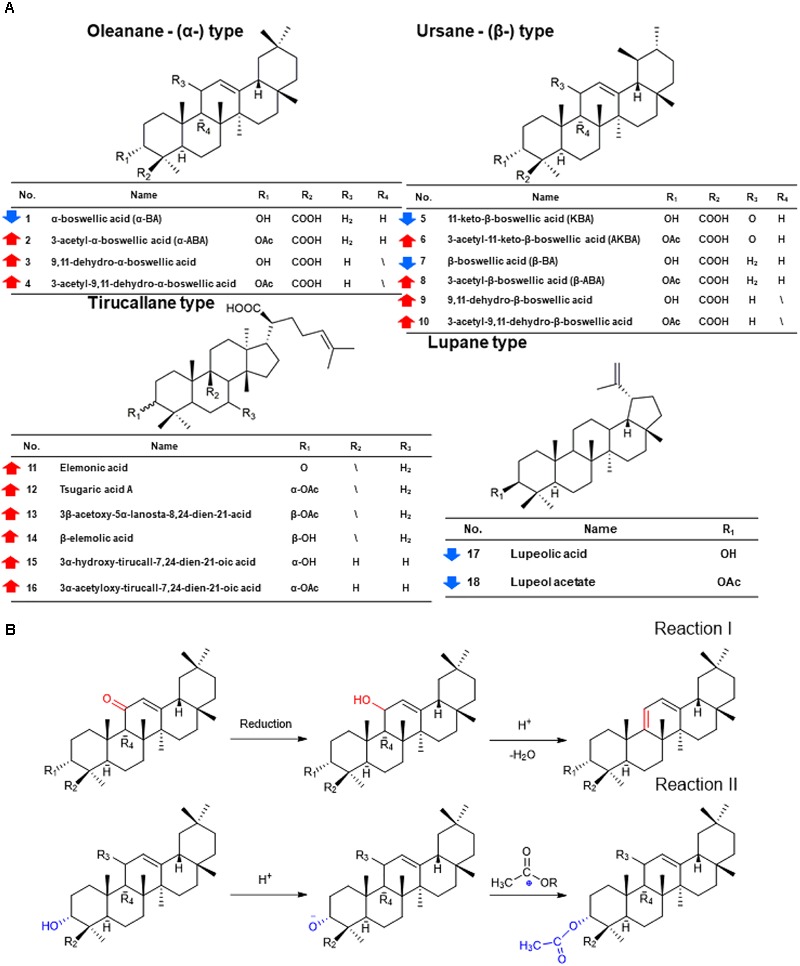
The chemical changes of frankincense and processed frankincense and the underlying regulation of Paozhi-perturbed metabolic reactions. **(A)** Structures and content changes of 18 potential biomarkers of frankincense and processed frankincense (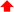 an increase; 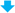 represents a decrease). **(B)** Two underlying regulations of Paozhi-perturbed metabolic reactions.

PCA and OPLS-DA were utilized to classify the metabolic phenotypes and identify the differentiating metabolites from the HSCCC fractions of frankincense and processed frankincense. A PCA score plot for the first and second principal components was utilized to depict the general variation among the samples (*R*^2^*X* = 0.851, *Q*^2^= 0.685), as shown in Figure 1C. It could divide the frankincense and processed frankincense samples into separate blocks. OPLS-DA was employed for classification or discrimination analyses. A variable importance plot (VIP plot) was utilized to identify the metabolites according to their contributions to the diverse clustering (Figure 1D). Among 153 detected compounds, 81 metabolites had VIP values greater than 1, and 54 of the compounds were identified. The identified metabolites are marked in the TIC chromatography in Supplementary Figure S2. The details of the identified compounds are summarized in Table 1. In total, 18 separated compounds were included in the identified metabolomics, and they were proven to make contributions to classifying the frankincense and processed frankincense. The 18 separated compounds consist of oleanane-(α)-type, ursane-(β)-type, tirucallane-type and lupine-type compounds, depending on their skeleton structures. As shown in Figure 2A, the compounds of tirucallane-type clearly occurred at high levels in the processed frankincense, while the compounds of lupine-type were found to be significantly higher in unprocessed samples. For oleanane-(α)-type and ursane-(β)-type compounds, 3-acetyl-9,11-dehydro-α-boswellic acid, 3-acetyl-9,11-dehydro-β-boswellic acid, 9,11-dehydro-α-boswellic acid and 9,11-dehydro-β-boswellic acid were increased in the processed frankincense, while, KBA and AKBA were found to be at higher levels in frankincense.

**Table 1:** Components identified in frankincense and processed frankincense.

No.	Compounds	Retention time	Molecular formula	Negative mode	Positive mode
1	3-Oxoazukisapogenol	9.984	C30H46O4	[M-H]^-^= 469.3260	[M+Na]^+^= 493.3310
2	3β-Acetoxyurs-18-ene	10.525	C32H52O2	[M-H]^-^= 467.3130	[M+H] = 469.3302
3	27,28-Dinorursane changyediyuine III	10.553	C29H40O5	[M-H]^-^= 467.3072	[M+Na]^+^= 491.3127
4	23-Norursane	11.303	C29H44O5	∖	[M+Na]^+^= 495.3412
5	Aceriphyllic acids E	11.721	C30H48O4	∖	[M+Na]^+^= 495.3503
6	Aceriphyllic acids F	12.239	C33H52O4	[M-H]^-^= 511.3341	∖
7	Atricins A	12.446	C31H46O4	[M-H]^-^= 469.3289	[M+Na]^+^= 493.3417
8	3,4-Di-epigypsogenin	12.591	C31H46O4	[M-H]^-^= 469.3083	[M+Na]^+^= 493.3316
9	Aceriphyllic acids E	13.041	C30H48O4	∖	[M+Na]^+^= 387.2555
10	KBA	13.830	C30H46O4	[M-H]^-^= 469.3277	[M+Na]^+^= 493.3378
11	Eleganenes B	14.281	C30H46O4	[M-H]^-^= 469.3113	[M+Na]^+^= 493.3378
12	28-Hydroxy-3-oxofriedelan-29-oic acid	14.654	C30H48O4	[M-H]^-^ = 471.3405	[M+Na]^+^= 495.3454
13	3β,6β-Dihydroxyurs-12-en-27-oic acid	15.376	C30H48O4	[M-H]^-^= 471.3421	[M+Na]^+^= 495.3409
14	3β,24-Dihydroxy-12-en-27-oic acid	15.537	C30H48O4	[M-H]^-^= 471.3432	[M+Na-H_2_O]^+^= 477.3371
15	3,4-Di-epigypsogenin	15.987	C31H46O4	[M-H]^-^= 469.3303	[M+Na]^+^= 493.3378
16	2α,3α-Dihtdroxy-urs-12-en-24-oic acid	16.180	C30H48O4	[M-H]^-^= 471.3422	[M+Na]^+^= 495.3491
17	3-Acetyl-11-hydroxy-β-boswellic acid	16.892	C32H50O5	[M-H]^-^= 513.3493	[M+Na]^+^= 537.3602
18	AKBA	16.937	C32H48O5	[M-H]^-^= 511.3372	[M+Na]^+^= 535.3518 [M+H]^+^= 513.3725
19	3-Acetyl-28-hydroxy-lupeolic acid	17.420	C30H48O4	[M-H]^-^= 513.3533	[M+Na]^+^= 537.3544
20	3β-Acetoxy-5α-lanosta-8,24-dien-21-acid	17.92	C30H48O4	[M-H]^-^= 513.3538	[M+Na]^+^= 537.3500
21	3α-Acetyloxy-tirucall-7,24-dien-21-oic acid	18.03	C30H48O3	[M-H]^-^= 513.3538	[M+Na]^+^= 537.3642
22	24-Norursane acetate	18.580	C31H48O2	[M-H]^-^= 451.3193	[M+Na]^+^= 475.3161 [M+H]^+^= 453.3219
23	12α-Hydroxyfriedelane-3,15-dione	19.175	C30H48O3	[M-H]^-^= 455.3476	[M+Na]^+^= 479.3495 [M+H]^+^= 457.3660
24	α-BA	19.726	C30H46O3	[M-H]^-^= 453.3348	[M+Na]^+^= 477.3372 [M+H]^+^= 455.3541
25	Elemonic acid	19.838	C30H46O3	[M-H]^-^= 453.3367	[M+Na]^+^= 477.3310
26	Elemolic acid	19.982	C30H48O3	[M-H] ^-^= 455.3520	[M+Na]^+^= 479.3572
27	Lupeolic acid	20.427	C30H48O3	[M-H]^-^= 455.3529	[M+Na]^+^= 479.3572
28	3β,22α-Dihydroxytaraxast-20-en-30-al	20.353	C31H50O2	[M-H]^-^= 453.3280	[M+Na]^+^= 477.3319
29	9,11-Dehydro-α-boswellic acid	20.563	C30H46O3	[M-H]- = 453.3643	[M+Na]^+^= 477.3223
30	3β-Hydroxytaraxasta-12,18-dien-28-oic acid	20.675	C30H46O3	[M-H]^-^= 453.3308	[M+Na]^+^= 477.3344
31	Masticadienonic acid	20.766	C30H48O3	[M-H]^-^= 455.3538	[M+Na]^+^= 479.3529
32	Ursane-3β,13α,18β-triol	20.77	C30H52O3	∖	[M+H]^+^= 483.3770
33	3α-Hydrotirucall-7,24-dien-21-oic acid	21.421	C30H46O3	[M-H]^-^= 453.3244	[M+Na]^+^= 477.3412 [M+H]^+^= 455.3231
34	9,11-Dehydro-β-boswellic acid	21.438	C30H46O3	[M-H]- = 453.3634	[M+Na]^+^= 477.3124
35	3β-Urs-12-en-29-oic acid	21.494	C30H48O3	[M-H]- = 455.3529	[M+Na]^+^= 479.3515
36	Taraxer-14-ene-1α,3β-diol-1-ketone	21.844	C30H48O2	∖	[M+Na]^+^= 463.3588
37	β-BA	22.122	C30H48O3	[M-H]- = 455.3529	∖
38	Tsugaric acid	22.725	C32H50O4	[M-H]^-^= 497.3681	[M+Na]^+^= 521.3586
39	11α-Methoxyurs-12-en-3-one	24.024	C31H50O2	∖	[M+Na]^+^= 517.3282
40	11β-Hydroxyfriedelan-3-one	25.214	C30H50O2	∖	[M+Na]^+^= 465.3766
41	21β-Hydroxyolean-12-en-3-one	25.561	C30H48O2	∖	[M+Na]^+^= 463.3571
42	Aceriphyllic acids C	25.656	C32H50O4	[M-H]^-^= 497.3571	[M+Na]^+^= 521.3642
43	Aceriphyllic acids H	26.360	C32H50O4	[M-H]^-^= 497.3637	[M+Na]^+^= 521.3590
44	3-Acetyl-9,11-dehydro-α-boswellic acid	26.638	C32H48O4	[M-H]^-^= 495.3396	[M+Na]^+^= 519.3454
45	3β-Hydroxytaraxasta-12,18-dien-28-oic acid	27.306	C30H46O3	[M-H]^-^= 453.3408	[M+Na]^+^= 477.3406
46	3-Acetyl-9,11-dehydro-β-boswellic acid	27.519	C32H48O4	[M-H]^-^= 495.3470	[M+Na]^+^= 519.3444
47	α-ABA	27.998	C32H50O4	[M-H]^-^= 497.3649	[M+Na]^+^= 521.3581
48	3β-Hydroxyglutin-5-en-28-oic acid	28.546	C30H48O3	[M-H]^-^= 455.3562	[M+Na]^+^= 479.3581
49	β-ABA	28.775	C32H50O4	[M-H]^-^= 497.3621	[M+Na]^+^= 521.3655
50	Aceriphyllic acids D	29.577	C32H50O3	∖	[M+Na]^+^= 505.3708 [M+Na-Ac]^+^= 463.3608
51	α-Amyrin	31.654	C30H50O	∖	[M+Na]^+^= 449.3753 [M+H]^+^= 427.3934
52	24-Norursa-3,12-dien-11-one	32.418	C29H44O	[M-H]^-^= 437.3304	[M+Na]^+^= 431.3345 [M+H]^+^= 409.3517
53	β-Anyrin	33.232	C30H50O	∖	[M+Na]^+^= 449.3753 [M+H]^+^= 427.3934
54	Roburic acid	34.025	C30H48O2	[M-H]^-^= 455.3538	[M+Na]^+^= 479.3537

For the structural identification of natural products, a couple of efficient ion data processing strategies based on using tandem mass spectrometry to rapidly profile the chemical constituents of complicated herbal extracts are beneficial to compound discovery (Qiao et al., 2016), performed according to reference substances and comparing the retention times, UV-visible spectra and mass characteristics with those in previous literature (Zhang C. et al., 2015). The accurate mass measurements of UHPLC-Qtof-MS give the elemental composition of parent and fragment ions used for identification. In addition, the chromatographic behaviors of some boswellic acids in the literature were utilized as complementary data for the identity confirmation of isomers. The number and types of the functional groups present on the triterpenic skeleton have, of course, a great influence on the retention time of these compounds. Concerning the role played by their chemical skeletons in the retention mechanism, ursane standards (β-BA or β-ABA) were always retained longer than were oleanane isomers (α-BA or α-ABA) and even longer than were lupine isomers. C-3 was proven to be an important criterion of retention. The β-configuration of C-3 always gave a longer retention time than did the α-configuration. Compounds of the same family were always eluted according to the following order: 3α-alcohols with a carboxylic functional group at C-24, the corresponding O-acetates, 3-ketones, 3α-alcohols and, finally, 3β-alcohols. Amyrins were not easily characterized because they were present at a very low intensity. 9,11-Dehydro derivatives of α-BA and β-BA and their corresponding acetylated forms were detected as characteristic components of processed frankincense. However, MS yields ambiguous fragmentation patterns for isomers. Therefore, NMR spectroscopy of the identified compounds was used to obtain complementary information to resolve their molecular configuration. Although TIC exhibited only a few peaks in UHPLC-Qtof-MS, a number of minor isomer compounds could be observed in SIM mode. More isomers could be identified by their chromatography behavior and NMR spectroscopy characteristics. The NMR spectral characteristics of compounds separated by HSCCC combined with PHPLC play a significant role in isomer identification.

Moreover, the larger lab-scale preparation of principal compounds lent to the bioactivity evaluation assay. The combination achieved the enrichment of minor concentrated metabolites from the crude extracts, which enabled detection of more minor and trace metabolites.

#### Semiquantitative Analysis and Potential Reactions During Processing

Using a semiquantitative assay, the content differences between 18 separated triterpenoids in the five batches of frankincense and processed frankincense samples could be evaluated. With respect to the semiquantitation of frankincense, the precursor ions of the characteristic components in 10 samples were extracted, and the absolute peak areas of a total of 18 triterpenoids were obtained. While the processing mechanism has remained a black box, several chemical translations during processing have been discovered, and structural variation in the present compounds and the positions of keto and acetyl groups are the most critical pathways for the formation of characteristic metabolites during processing. As shown in Figure 2A, the compounds with 9,11-dehydro structures were increased after processing, while the compounds with 11-keto structures were decreased after processing. Regarding the compounds with 11-keto structures, temperature has an important effect on the biosynthesis and accumulation of 11-hydroxy-structured boswellic acid during the processing procedure through the degradation of the natural compound 11-hydroxy-boswellic acid into the thermodynamically more stable product. In addition, 11-methoxy boswellic acid may be the metastable intermediate of this conversion (Schweizer et al., 2000) because the 11-keto group is a protophilic center for the absorption of protons. It was indicated that structural variation in the position of the keto group occurred during processing in reaction I (as shown in Figure 2B). For α/β boswellic acids with 3-OH, processing decreases their contents. In contrast, the content of 3-acetyl-α/β boswellic acids in processed frankincense were higher than those in frankincense. It was believed that processing may esterify the 3-OH of α/β boswellic acid, which is exhibited in reaction II in Figure 2B. Moreover, lupeolic acid and lupeol acetate were decreased after processing. In addition, the contents of six compounds of tirucallane type (elemonic acid, tsugaric acid A, 3β-acetary-5α-lanosta-8,24-dien-21-acid, β-elemolic acid, 3α-hydrotirucall-7,24-dien-21-oic acid and 3α-acetyloxy-tirucall-7,24-dien-21-oic acid) were increased after processing. Their chemical reactions can be investigated in further research.

### The Prediction of Absorption and Pharmacological Properties

To establish the relationship between the changes of compounds, absorption and cure efficiency, a network construction approach was used to predict the processing effects in this paper. Absorption was defined as the process by which the drug molecule moves from the site of administration into the system. Oral administration is the most preferred drug-delivery method. The dissolution and ability to permeate into the gastrointestinal tract are considered to be the major barriers to drug availability (Honório et al., 2013). OB is considered a key parameter in evaluating the absorption effect. Meanwhile, Caco-2 cell permeability was also a significant standard for evaluating oral absorption.

**FIGURE 3:**
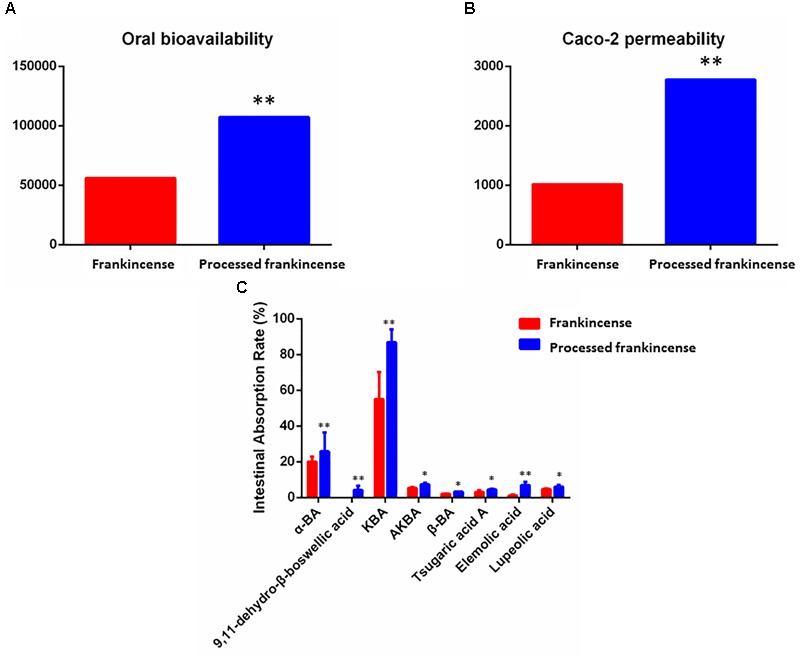
Absorption prediction and validation of frankincense and processed frankincense. **(A)** OB predictive parameters of frankincense and processed frankincense. **(B)** Caco-2 predictive parameters of frankincense and processed frankincense. **(C)** Intestinal absorption rate of compounds in frankincense and processed frankincense. (^∗^ and ^∗∗^*P* < 0.05 and 0.01, comparison with the frankincense group).

In the current work, 18 potential biomarkers attributed to frankincense and processed frankincense were predicted by two absorption-related models, including the OB and Caco-2 permeability values (as shown in Table 2). With respect to the absorption prediction of frankincense and processed frankincense causing problems with the varied content of multiple attribute compounds, the WI value of each compound was calculated. Single-compound absorption properties, weight indices and contents were taken consideration to evaluate the absorption properties of frankincense and processed frankincense. As shown in Figures 3A,B, an increase of both OB and Caco-2 permeability values occurred after processing. This indicated that processing may play an important role in enhancing the absorption of the compounds. Whether processing could contribute to bioavailability improvement should be further affirmed to elaborate the effects of processing.

**Table 2:** Absorption prediction parameters of 18 potential biomarkers.

	Content in frankincense (μg/g)	Content in processed frankincense (μg/g)	OB value	Caco-2 permeability value	WI value
α-BA	25737.00	10847.00	12.53	0.62	0.025
α-ABA	2465.00	4353.00	11.02	0.72	0.012
9,11-Dehydro-α-boswellic acid	130.74	3497.77	42.86	0.55	0.161
3-Acetyl-9,11-dehydro-α-boswellic acid	110.67	5326.00	11.09	0.55	0.177
KBA	53741.91	20339.34	39.60	0.52	0.031
AKBA	5605.78	3184.30	38.71	0.48	0.011
β-BA	1012.78	563.91	39.13	0.53	0.012
β-ABA	3476.00	5476.00	40.11	0.55	0.008
9,11-Dehydro-β-boswellic acid	130.74	3497.77	75.45	-0.98	0.161
3-Acetyl-9,11-dehydro-β-boswellic acid	110.67	5326.00	9.98	1.40	0.177
Elemonic acid	5756.00	8654.00	11.11	0.60	0.006
Tsugaric acid A	63.93	387.60	33.88	0.65	0.085
3β-Acetoxy-5α-lanosta-8,24-dien-21-acid	2543.00	4521.00	39.87	0.72	0.012
β-Elemolic acid	3447.24	998.91	17.73	0.21	0.048
3α-Hydroxy-tirucall-7,24-dien-21-oic acid	3345.00	5421.00	17.75	0.16	0.008
3α-Acetyloxy-tirucall-7,24-dien-21-oic acid	1234.00	3267.00	39.55	0.63	0.032
Lupeolic acid	2134.00	1237.00	10.01	0.53	0.011
Lupeol acetate	324.00	145.00	17.75	0.92	0.022
Frankincense	∖	∖	56125.24	1019.11	∖
Processed frankincense	∖	∖	107359.64	2779.01	∖

**FIGURE 4:**
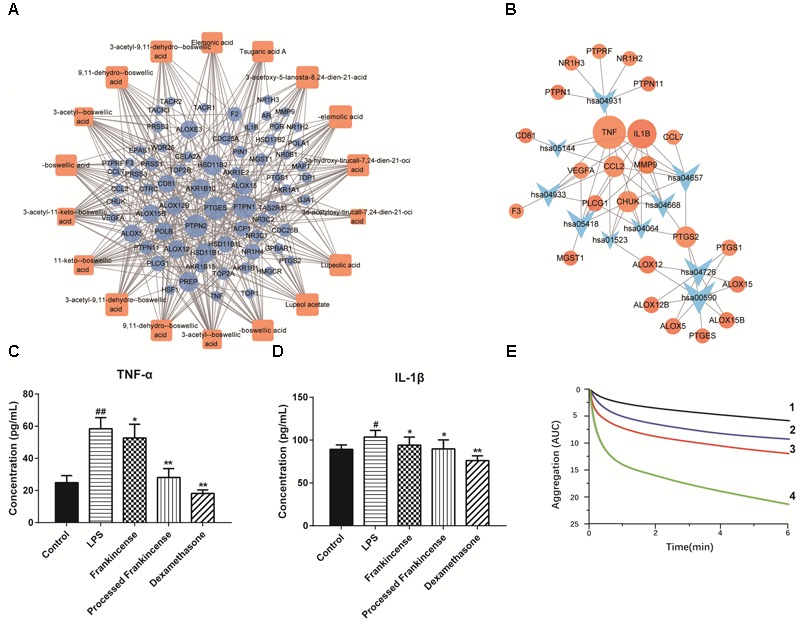
Pharmacological prediction and validation of frankincense and processed frankincense. **(A)** Potential biomarker-target network of frankincense and processed frankincense. **(B)** Target-pathway of potential biomarkers of frankincense and processed frankincense. **(C)** Effects of frankincense and processed frankincense on TNF-α. **(D)** Effects of frankincense and processed frankincense on IL-1β. **(E)** Effects of frankincense and processed frankincense on antiplatelet effects induced by AA (1. Aspirin group; 2. Processed frankincense group; 3. Frankincense group; 4. Control group). (^##^*P* < 0.01, comparison with the control group; ^∗^ and ^∗∗^*P* < 0.05 and 0.01, comparison with the LPS group).

To explore the potential therapeutic mechanisms of frankincense and processed frankincense, 18 potential biomarkers and 69 targets (Supplementary Table S2) were used to construct the C-T network (Figure 4A). Several of these potential biomarkers are related to multiple targets, resulting in 349 component-target associations. AKBA (degree = 28) has the highest number of targets, followed by KBA (degree = 26), β-ABA (degree = 25), α-ABA (degree = 24), β-BA (degree = 21), and α-BA (degree = 21), demonstrating the crucial roles of these components. Interestingly, multiple targets mediated by 18 potential biomarkers from frankincense and processed frankincense are involved in inflammatory responses. For example, AKBA may have interactions with 28 targets, including ALOX5, CHUK, IL1B, PTGES, PTPN1, PTPN2, etc. Actually, AKBA has been identified as a potent inhibitor of ALOX5 (5-LO), an enzyme responsible for inflammation (Siddiqui, 2011). Beyond that, it can also exert anti-inflammatory effects by directly interacting with IκB kinases (CHUK) (Syrovets et al., 2005) and inhibiting IL1B (Gayathri et al., 2007).

Furthermore, the canonical pathways associated with the targets were extracted from the Kyoto Encyclopedia of Genes and Genomes (KEGG^3^) database, which yielded 10 KEGG pathways (*p* < 0.02, *q* < 0.02), including AA metabolism, the IL-17 signaling pathway, serotonergic synapses, insulin resistance, the TNF signaling pathway, the NF-kappa B signaling pathway, etc. (Supplementary Figure S1). To elaborate on the involved significant pathways of frankincense and processed frankincense, the target proteins were mapped on the KEGG pathways, resulting in a T-P network (Figure 4B). The T-P network contains 34 nodes (10 pathways and 24 targets and 60 edges). The AA metabolism (hsa00590) pathway exhibits the highest number of target connections (degree = 8), followed by the IL-17 signaling pathway (hsa04657, degree = 7) and serotonergic synapses (hsa04726, degree = 7). Based on the results, it was found that these high-degree pathways were closely related to inflammation. Particularly, the crucial AA metabolism can produce a variety of products which mediate or modulate inflammatory reactions (Samuelsson, 1991) and is regulated by eight potential targets (PTGES, PTGS1, PTGS2, ALOX5, ALOX15, etc.). In addition, the IL-17 signaling pathway can also play crucial roles in both acute and chronic inflammatory responses (Qian et al., 2010). The NF-kappa B signaling pathway is related to various inflammatory diseases (Liu et al., 2017). Consequently, the above discussions indicate that frankincense and processed frankincense have anti-inflammatory therapeutic effects through acting on genes involved in multiple inflammatory pathways.

### The Validation of Absorption and Pharmacological Effects

#### The Validation of Absorption Predictions

As the result of absorption prediction, processing was believed to have a positive effect on frankincense absorption. To confirm this idea, there is an urgent need for validation. Gastrointestinal permeability is a fundamental parameter determining oral drug absorption in oral administration, and the rat everted gut-sac model is a classical model utilized to investigate intestinal absorption (Takada et al., 1997). In the present research, the contents of compounds in the inner solution of everted gut-sacs were determined by the established UHPLC-QqQ-MS method (shown in Supplementary Table S3 and Supplementary Figure S3).

The procedure of optimizing the MRM transitions for all target analytes was as follows. The precursor and product ions were determined by standard solutions in scan and product ion modes. The fragmentor energy and collision energy parameters were further optimized based on the abundance of precursor and product ions (as shown in Supplementary Table S3). Representative MRM chromatograms and total ion chromatograms are shown in Supplementary Figure S3. Higher contents represent ideal intestinal permeability. As shown in Figure 3C, only 8 of 18 potential biomarkers could be detected in the inner solution. The reason may be the unsatisfactory bioavailability of boswellic acids (Du et al., 2015). The contents of the eight compounds were higher in processed frankincense than in frankincense. 9,11-Dehydro-β-boswellic acid showed a rapid rise after processing. These results indicated that the components of frankincense can increasingly be transported from the medium into the serosal fluid of the gut sacs across the intestinal barrier after processing. This finding validated the prediction that the processing procedure successfully improved the absorption of compounds. The enhancement of bioavailability after processing has been published previously (Cui et al., 2015; Zhao et al., 2016). However, innovative drug availability research is known for requiring big investments, high risk and long development times, as well as for facing big challenges. Recent advances have been made in the development of models to assess and predict intestinal absorption properties in the early stages of drug investigation, addressing challenges, limitations and opportunities in medicinal chemistry (Honório et al., 2013). The combination of absorption properties prediction and confirmation assays in the present strategy may raise the efficiency of absorption properties research.

#### The Validation of Pharmacological Data

As shown in the T-P network, TNF and IL-1β were among the top-ranking compounds and were linked by 8 and 7 pathways (Figure 4). LPS is a component of the outer membrane of Gram-negative bacteria that potently promotes the activation of macrophages and microglia cells (Schiavano et al., 2016). In this paper, LPS was selected as the stimulator, and the anti-inflammatory properties of frankincense and processed frankincense were evaluated by the levels of TNF-α and IL-1β. The results showed that the extracts of frankincense and processed frankincense (2–8 μg/mL) had no effect on the viability of cells. When stimulated by LPS, U937 cells released significantly increasing levels of TNF-α and IL-1β compared with untreated controls. As shown in Figures 4C,D, cells pretreated with frankincense and processed frankincense showed significant inhibition of TNF-α and IL-1β. Among the pretreated group, frankincense extraction decreased the level of TNF-α and IL-1β in cellular supernatants compared to the effects of untreated frankincense. This indicated that frankincense may harbor the strongest anti-inflammatory activities. As the results of the semiquantitative analysis study show, the compounds with 9,11-dehydro structures were increased after processing, while the compounds with 11-keto structures were decreased after processing. According to the anti-inflammation study, 3-acetyl-9,11-dehydro-BA almost abolished 5-lipoxygenase activity at an IC_50_ = 0.75 μM, while AKBA did so at an IC_50_ = 2.7 μM (Schweizer et al., 2000). This means that the anti-inflammation activity was enhanced after processing because the increased compounds have stronger inhibition effects toward inflammation, which is consistent with the predication of the anti-inflammatory activities through network pharmacology. The data suggested that the chemical variation between frankincense and processed frankincense could seriously influence their potential therapeutic effectiveness and the potency of their biological actions. To further elucidate the anti-inflammatory mechanisms of the processing procedure of frankincense, the delineated functional pathways will be deeply studied in the future.

Additionally, AA and its metabolites in AA metabolism are significant precursors of various bioactive molecules in the cardiovascular system (Kiso, 2011). They have been shown to modulate platelet aggregation, vasorelaxation and vasoconstriction (Yao et al., 2015). An aggregometry assay induced by AA was performed. The results of AA-induced aggregometry measurement in whole blood are presented in Figure 4E. The test successfully demonstrated the anti-platelet effect of frankincense and processed frankincense against AA-induced platelet aggregation responses. Treatment with frankincense and processed frankincense metabolites could also significantly decrease the response but with a less potent effect in comparison to processed frankincense.

## Conclusion

**FIGURE 5:**
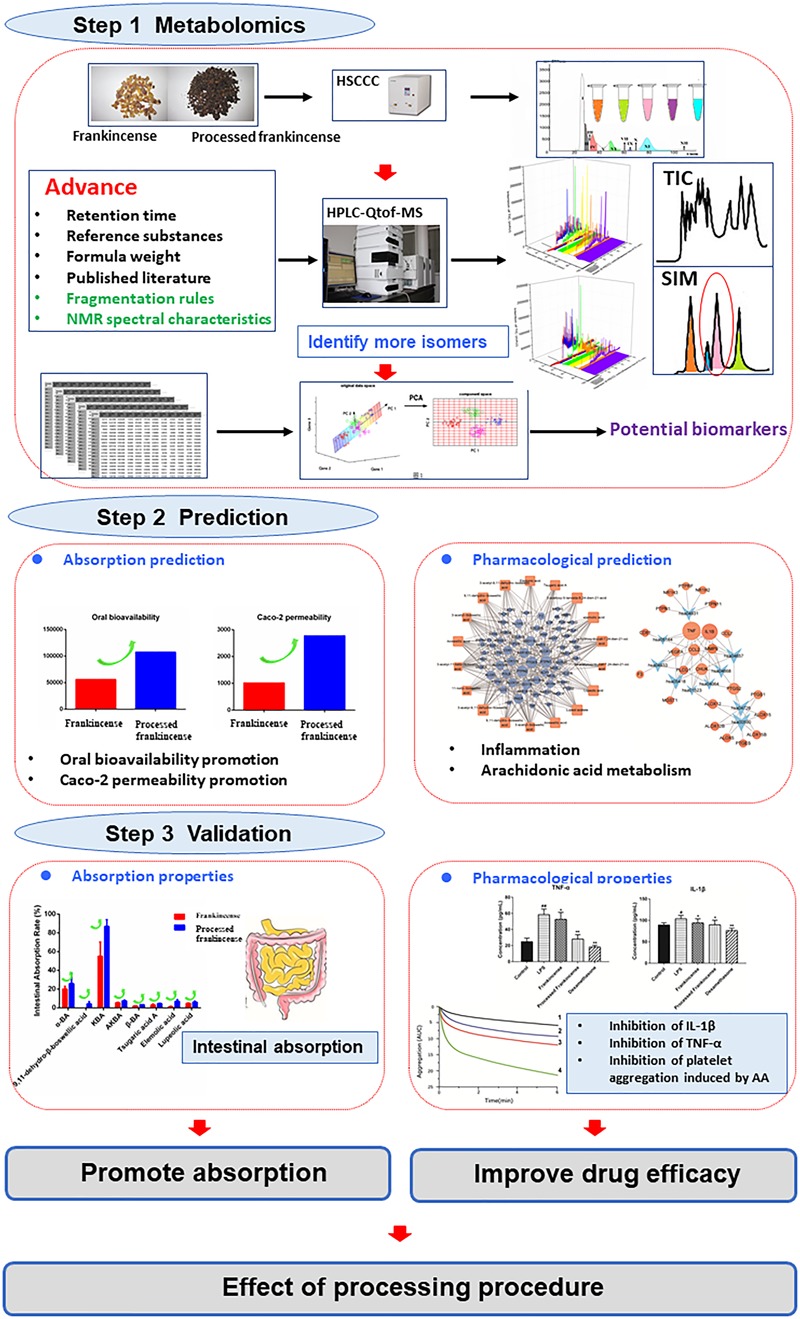
Description of the strategy.

The aim of this study was to establish a strategy for realizing the investigation of the effects of processing procedures by comparing the global metabolites, (as shown in Figure 5) absorption and pharmacological properties. Because of the limit of mass spectrometry in the analysis of isomers, an improved metabolomics method was put forward. HSCCC realized both fractions of the principal compounds and the concentrations of minor metabolites. Additionally, pure compounds were obtained in this strategy. A comprehensive metabolomics analysis conducted by UHPLC-Qtof-MS coupled with multivariate statistics was performed to entirely characterize the chemical components and to discover the potential biomarkers between frankincense and processed frankincense. Total methanol extracts and HSCCC fractions of frankincense and processed frankincense were analyzed. The results showed that compared to the methanol extract, more compounds can be detected in the HSCCC fractions. The combination of HSCCC and UHPLC-Qtof-MS provides a powerful technique for the global chemical profiling of complex matrices. NMR spectroscopies of the drawn compounds were used to obtain complementary information to resolve their molecular configurations. In total, 81 metabolites were selected as potential biomarkers among the 153 detected compounds for their VIP values of greater than one. Of these, 54 compounds, including the 18 separated compounds, were identified successfully, and they were proven to contribute to classifying the frankincense and processed frankincense. Then, a holistic quantitative evaluation with multistatistical analysis of the specific potential biomarkers was conducted to elaborate the regulation of processing-perturbed metabolic pathways. Harboring much potential to interpret the mechanisms of processing at the molecular-network level, network pharmacology screening was utilized to predict the absorption and pharmacological properties. This indicated that processing can promote absorption and possibly improve the cure efficiency. Finally, the extracts of frankincense and processed frankincense were investigated for their intestinal absorption and biological activity for experimental validation. The experimental validation provided convincing evidence that the processing procedure leads to changes of the chemical metabolites, affecting the absorption and cure efficiency. The global changes of the metabolites and the absorption and pharmacological effects of processing were depicted in a systematic manner. Ultimately, this work might provide a feasible strategy for the discrimination of different processed products in a systematic manner and facilitate a better understanding of their different medicinal uses.

## Author Contributions

AL and ZL provided the concept and designed the study. ZN, CW, and YL conducted the experiments and wrote the manuscript. ZN, CW, YL, ZS, XM, and DL participated in the experiments. AL and ZL contributed to revising and proofreading the manuscript. All authors read and approved the final manuscript.

## Conflict of Interest Statement

The authors declare that the research was conducted in the absence of any commercial or financial relationships that could be construed as a potential conflict of interest.

## Footnotes

1 http://lsp.nwu.edu.cn/tcmsp.php

2 https://www.bioconductor.org/

3 http://www.genome.jp/kegg/

## Supplementary Material

FIGURE S1 The barplot of canonical pathways associated with the targets.

FIGURE S2 The TIC chromatography of frankincense and processed frankincense.

FIGURE S3 MRM figure for seven compounds in the inner solution of frankincense and processed frankincense.

Table S1 Partition coefficient (*K*) values of compounds in different solvent systems.

Table S2 The information of the related targets of frankincense.

Table S3 The information for MRM parameters.
