# Supplementary material for: Integrating Strategies of Herbal Metabolomics, Network Pharmacology, and Experiment Validation to Investigate Frankincense Processing Effects
Source: Front Pharmacol. 2018 Dec 18;9:1482. doi: 10.3389/fphar.2018.01482 (PMC6305425; doi:10.3389/fphar.2018.01482)
Supplement: FIGURE S1 — The barplot of canonical pathways associated with the targets. [file Data_Sheet_1.ZIP › revise supplementary material/Table S1 The partition coefficient.docx]

Table S1 Partition coefficient (K) values of compounds in different solvent systems

|  | 11-keto-β-boswellic acid | 3-acetyl-11-keto-β-boswellic acid | α-boswellic acid | β-boswellic acid | 3-acetyl-α-boswellic acid | 3-acetyl-β-boswellic acid |
| --- | --- | --- | --- | --- | --- | --- |
| *n-heptane-methyl acetate-acetonitrile-water (4:4:3:4) Upper layer/Middle layer | 0.15 | 0.25 | 0.61 | 0.81 | 1.10 | 1.04 |
| *n-heptane-acetonitrile-dichloromethane-H_2_O (5:5:1:5) Upper layer/Middle layer | 0.04 | 0.05 | 0.18 | 0.16 | 0.40 | 0.43 |
| n-heptane-acetonitrile-ethyl acetate (5:5:1) | 0.05 | 0.10 | 0.37 | 0.45 | 0.84 | 0.90 |
| n-heptane-ethyl acetate-methanol-H_2_O (6:1:6:1) | 0.70 | 0.47 | 0.48 | 0.56 | 3.14 | 3.11 |
| n-heptane-ethyl acetate-methanol-H_2_O (3:2:3:2) | 1.56 | 7.27 | 15.75 | 17.06 | 54.98 | 48.03 |

* Three-phase solvent system
