# Supplementary material for: Integrating Strategies of Herbal Metabolomics, Network Pharmacology, and Experiment Validation to Investigate Frankincense Processing Effects
Source: Front Pharmacol. 2018 Dec 18;9:1482. doi: 10.3389/fphar.2018.01482 (PMC6305425; doi:10.3389/fphar.2018.01482)
Supplement: FIGURE S1 — The barplot of canonical pathways associated with the targets. [file Data_Sheet_1.ZIP › revise supplementary material/Table S2 69 targets of 18 potential biomarkers.docx]

Table S2 The information of the related targets of frankincense

| **Gene** | **Protein name** | **Uniprot ID** |
| --- | --- | --- |
| ACP1 | Low molecular weight phosphotyrosine protein phosphatase | P24666 |
| AKR1A1 | Alcohol dehydrogenase [NADP(+)] | P14550 |
| AKR1B1 | Aldose reductase | P15121 |
| AKR1B10 | Aldo-keto reductase family 1 member B10 | O60218 |
| AKR1B15 | Aldo-keto reductase family 1 member B15 | C9JRZ8 |
| AKR1E2 | 1,5-anhydro-D-fructose reductase | Q96JD6 |
| ALOX12 | Arachidonate 12-lipoxygenase, 12S-type | P18054 |
| ALOX12B | Arachidonate 12-lipoxygenase, 12R-type | O75342 |
| ALOX15 | Arachidonate 15-lipoxygenase | P16050 |
| ALOX15B | Arachidonate 15-lipoxygenase B | O15296 |
| ALOX5 | Arachidonate 5-lipoxygenase | P09917 |
| ALOXE3 | Hydroperoxide isomerase ALOXE3 | Q9BYJ1 |
| AR | Androgen receptor | P10275 |
| CASP9 | Caspase-9 | P55211 |
| CCL2 | C-C motif chemokine 2 | P13500 |
| CCL7 | C-C motif chemokine 7 | P80098 |
| CD81 | CD81 antigen | P60033 |
| CDC25A | M-phase inducer phosphatase 1 | P30304 |
| CDC25B | M-phase inducer phosphatase 2 | P30305 |
| CELA2A | Chymotrypsin-like elastase family member 2A | P08217 |
| CHUK | Inhibitor of nuclear factor kappa-B kinase subunit alpha | O15111 |
| CTRC | Chymotrypsin-C | Q99895 |
| EPAS1 | Endothelial PAS domain-containing protein 1 | Q99814 |
| F2 | Prothrombin | P00734 |
| F3 | Tissue factor | P13726 |
| GJA1 | Gap junction alpha-1 protein | P17302 |
| GPBAR1 | G-protein coupled bile acid receptor 1 | Q8TDU6 |
| HMGCR | 3-hydroxy-3-methylglutaryl-coenzyme A reductase | P04035 |
| HSD11B1 | Corticosteroid 11-beta-dehydrogenase isozyme 1 | P28845 |
| HSD11B1L | Hydroxysteroid 11-beta-dehydrogenase 1-like protein | Q7Z5J1 |
| HSD11B2 | Corticosteroid 11-beta-dehydrogenase isozyme 2 | P80365 |
| HSD17B2 | Estradiol 17-beta-dehydrogenase 2 | P37059 |
| HSF1 | Heat shock factor protein 1 | Q00613 |
| IL1B | Interleukin-1 beta | P01584 |
| MAPT | Microtubule-associated protein tau | P10636 |
| MGST1 | Microsomal glutathione S-transferase 1 | P10620 |
| MMP9 | Matrix metalloproteinase-9 | P14780 |
| NR0B1 | Nuclear receptor subfamily 0 group B member 1 | P51843 |
| NR1H2 | Oxysterols receptor LXR-beta | P55055 |
| NR1H3 | Oxysterols receptor LXR-alpha | Q13133 |
| NR1H4 | Bile acid receptor | Q96RI1 |
| NR3C1 | Glucocorticoid receptor | P04150 |
| NR3C2 | Mineralocorticoid receptor | P08235 |
| PGR | Progesterone receptor | P06401 |
| PIN1 | Peptidyl-prolyl cis-trans isomerase NIMA-interacting 1 | Q13526 |
| PLCG1 | 1-phosphatidylinositol 4,5-bisphosphate phosphodiesterase gamma-1 | P19174 |
| POLA1 | DNA polymerase alpha catalytic subunit | P09884 |
| POLB | DNA polymerase beta | P06746 |
| PREP | Prolyl endopeptidase | P48147 |
| PRSS1 | Trypsin-1 | P07477 |
| PRSS2 | Trypsin-2 | P07478 |
| PRSS3 | Trypsin-3 | P35030 |
| PTGES | Prostaglandin E synthase | O14684 |
| PTGS1 | Prostaglandin G/H synthase 1 | P23219 |
| PTGS2 | Prostaglandin G/H synthase 2 | P35354 |
| PTPN1 | Tyrosine-protein phosphatase non-receptor type 1 | P18031 |
| PTPN11 | Tyrosine-protein phosphatase non-receptor type 11 | Q06124 |
| PTPN2 | Tyrosine-protein phosphatase non-receptor type 2 | P17706 |
| PTPRF | Receptor-type tyrosine-protein phosphatase F | P10586 |
| TACR1 | Substance-P receptor | P25103 |
| TACR2 | Substance-K receptor | P21452 |
| TACR3 | Neuromedin-K receptor | P29371 |
| TAS2R31 | Taste receptor type 2 member 31 | P59538 |
| TDP1 | Tyrosyl-DNA phosphodiesterase 1 | Q9NUW8 |
| TNF | Tumor necrosis factor | P01375 |
| TOP1 | DNA topoisomerase 1 | P11387 |
| TOP2A | DNA topoisomerase 2-alpha | P11388 |
| TOP2B | DNA topoisomerase 2-beta | Q02880 |
| VEGFA | Vascular endothelial growth factor A | P15692 |
| WDR26 | WD repeat-containing protein 26 | Q9H7D7 |
