# Supplementary material for: Integrating Strategies of Herbal Metabolomics, Network Pharmacology, and Experiment Validation to Investigate Frankincense Processing Effects
Source: Front Pharmacol. 2018 Dec 18;9:1482. doi: 10.3389/fphar.2018.01482 (PMC6305425; doi:10.3389/fphar.2018.01482)
Supplement: FIGURE S1 — The barplot of canonical pathways associated with the targets. [file Data_Sheet_1.ZIP › revise supplementary material/Table S3 The information for MRM parameters.docx]

Table S3 The information for MRM parameters

| No. | Retention time | Compounds | Precursor ion | Product ion | Fragmentor | Collision energy |
| --- | --- | --- | --- | --- | --- | --- |
| 1 | 5.522 | 11-keto-β-boswellic acid | 469.4 | 391.5*, 407.4 | 160 | 30 |
| 2 | 9.92 | 3-acetyl-11-keto-β-boswellic acid | 511 | 255.4* | 115 | 11 |
| 3 | 9.758 | Elemolic acid | 455 | 339.4*, 424.9 | 270 | 45 |
| 4 | 10.372 | α- boswellic acid | 455 | 372.4, 437.5* | 270 | 43 |
| 5 | 11.86 | Luprolic acid | 455 | 409.4, 437.4* | 160 | 40 |
| 6 | 13.135 | Tsugaric acid | 497 | 437.4*, 467.3 | 260 | 45 |
| 7 | 13.774 | 9,11-dehydro-β-boswellic acid | 453 | 359.4, 375.5* | 190 | 40 |
| 8 | 15.589 | β- boswellic acid | 455 | 377.4*, 437.4 | 110 | 35 |

*Quantitative product ion
