# Supplementary figures and images for: Integrating Strategies of Herbal Metabolomics, Network Pharmacology, and Experiment Validation to Investigate Frankincense Processing Effects
Source: Front Pharmacol. 2018 Dec 18;9:1482. doi: 10.3389/fphar.2018.01482 (PMC6305425; doi:10.3389/fphar.2018.01482)

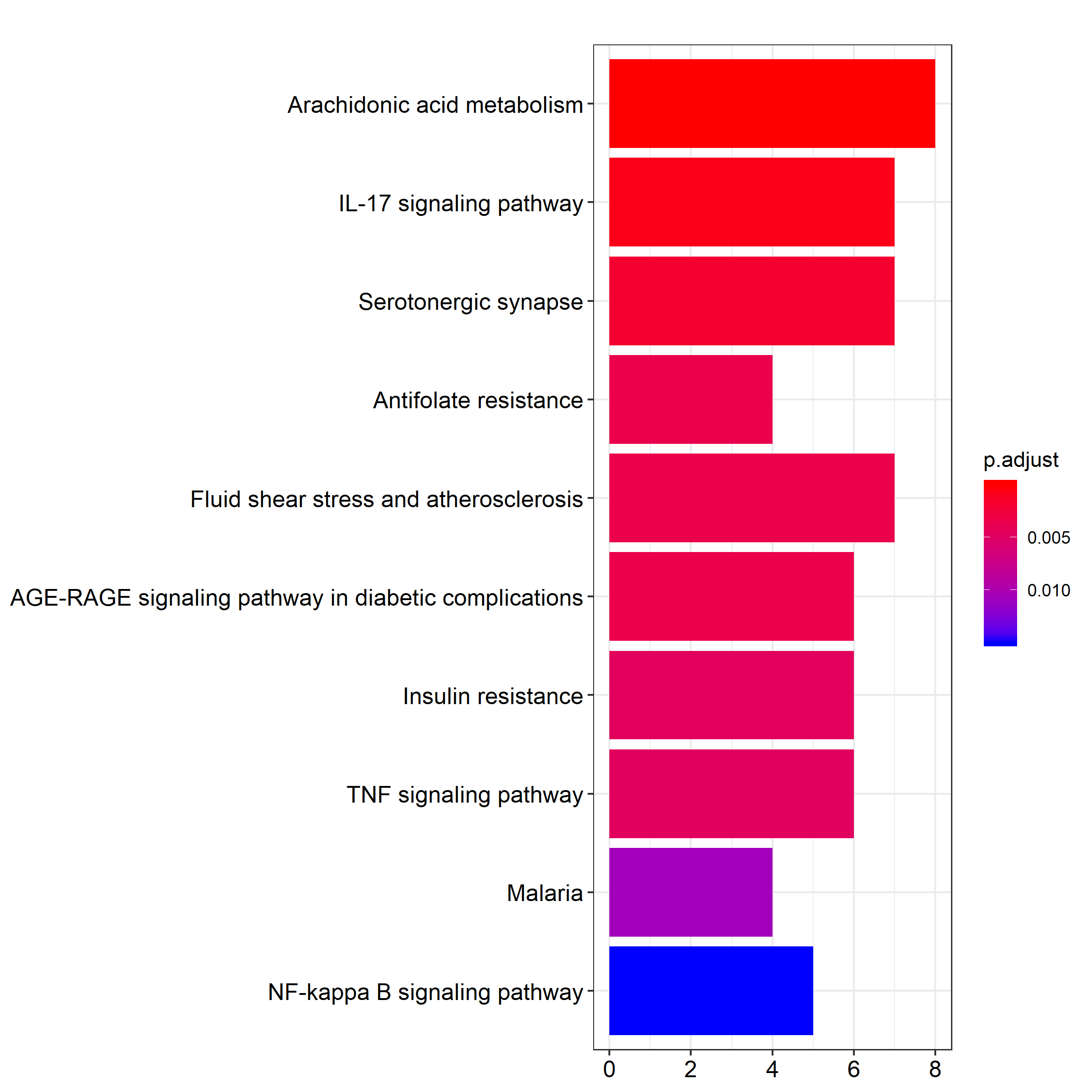

Supplement: FIGURE S1 — The barplot of canonical pathways associated with the targets. [file Data_Sheet_1.ZIP › revise supplementary material/Figure S1.tiff]

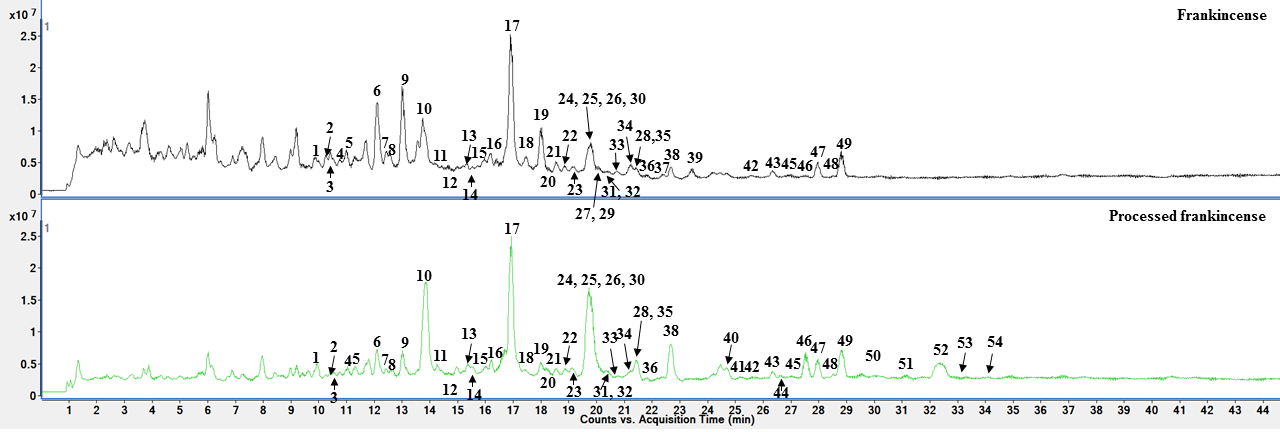

Supplement: FIGURE S1 — The barplot of canonical pathways associated with the targets. [file Data_Sheet_1.ZIP › revise supplementary material/Figure S2.tif]

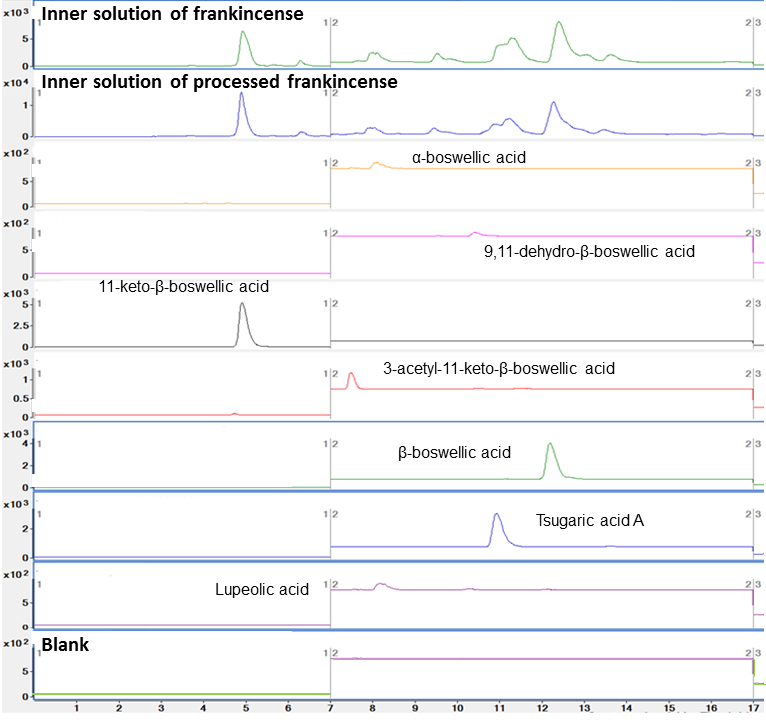

Supplement: FIGURE S1 — The barplot of canonical pathways associated with the targets. [file Data_Sheet_1.ZIP › revise supplementary material/Figure S3.TIF]
